# 3D-PAD: Paper-Based Analytical Devices with Integrated Three-Dimensional Features

**DOI:** 10.3390/bios11030084

**Published:** 2021-03-17

**Authors:** James S. Ng, Michinao Hashimoto

**Affiliations:** 1 Pillar of Engineering Product Development, Singapore University of Technology and Design, 8 Somapah Road, Singapore 487372, Singapore; james_ng@mymail.sutd.edu.sg; 2 SUTD-MIT International Design Centre, Singapore University of Technology and Design, 8 Somapah Road, Singapore 487372, Singapore; 3 Digital Manufacturing and Design Centre, Singapore University of Technology and Design, 8 Somapah Road, Singapore 487372, Singapore

**Keywords:** paper-based microfluidics, paper analytical device, 3D printing, microfabrication, digital fabrication

## Abstract

This paper describes the use of fused deposition modeling (FDM) printing to fabricate paper-based analytical devices (PAD) with three-dimensional (3D) features, which is termed as 3D-PAD. Material depositions followed by heat reflow is a standard approach for the fabrication of PAD. Such devices are primarily two-dimensional (2D) and can hold only a limited amount of liquid samples in the device. This constraint can pose problems when the sample consists of organic solvents that have low interfacial energies with the hydrophobic barriers. To overcome this limitation, we developed a method to fabricate PAD integrated with 3D features (vertical walls as an example) by FDM 3D printing. 3D-PADs were fabricated using two types of thermoplastics. One thermoplastic had a low melting point that formed hydrophobic barriers upon penetration, and another thermoplastic had a high melting point that maintained 3D features on the filter paper without reflowing. We used polycaprolactone (PCL) for the former, and polylactic acid (PLA) for the latter. Both PCL and PLA were printed with FDM without gaps at the interface, and the resulting paper-based devices possessed hydrophobic barriers consisting of PCL seamlessly integrated with vertical features consisting of PLA. We validated the capability of 3D-PAD to hold 30 μL of solvents (ethanol, isopropyl alcohol, and acetone), all of which would not be retained on conventional PADs fabricated with solid wax printers. To highlight the importance of containing an increased amount of liquid samples, a colorimetric assay for the formation of dimethylglyoxime (DMG)-Ni (II) was demonstrated using two volumes (10 μL and 30 μL) of solvent-based dimethylglyoxime (DMG). FDM printing of 3D-PAD enabled the facile construction of 3D structures integrated with PAD, which would find applications in paper-based chemical and biological assays requiring organic solvents.

## 1. Introduction

This paper describes the use of fused deposition modeling (FDM) printing to fabricate paper-based analytical devices (PAD) integrated with three-dimensional (3D) features. Direct material printing (such as solid wax printing and inkjet printing) followed by heating is a standard approach for the fabrication of PAD. Devices fabricated by such methods are inherently two-dimensional (2D); such conventional PADs are not able to hold organic solvents that have low interfacial energies with hydrophobic materials. To overcome this limitation, we fabricated PADs integrated with 3D features (such as vertical walls) by FDM 3D printing, which we termed 3D-PAD. 3D-PADs have improved the containment of organic solvents (e.g., 30 μL of ethanol, isopropyl alcohol (IPA), and acetone), which should find applications in handling solvent-based samples for paper-based chemical and biological assays.

Paper is an attractive substrate for point-of-care testing (POCT) due to its portability, cost, and ease of handling. Filter papers consist primarily of cellulose. They are disposable and widely used as a substrate for analytical devices. The porous nature of the cellulose papers permits handling and retaining of aqueous samples inside the matrices. Cellulose contains hydroxyl groups, and the surface of the cellulose could be readily functionalized to enhance the functionality of the device [1,2]. For example, colorimetric reagents can be immobilized to cellulose to report the presence of specific analytes. To this end, PADs have been developed for chemical sensing [3,4,5,6], environmental monitoring [7,8,9,10], and detection of microorganisms [11,12,13,14,15]. Fabrication of PAD was initially demonstrated via photolithography [16]. Over the past decade, alternative technologies were developed, including wax printing [17,18,19,20,21,22,23,24,25,26], inkjet printing [27,28], screen printing [29,30,31,32], and laser printing [33,34]. All of these approaches are based on the 2D patterning of materials. In brief, hydrophobic materials are patterned digitally or manually, and the treatment with heat (that promotes reflowing and curing) forms hydrophobic barriers across the entire thickness of the filter paper. The resulting devices are inherently planar and contained in a flat substrate. The patterned hydrophobic barriers permit the confinement of aqueous samples due to the high interfacial energy between water and wax, and aqueous samples can be enclosed in the regions where porous cellulose structures are exposed.

Despite the successful development of PADs, the 2D nature of PAD permits only a limited volume of liquid samples in the device. Breaching of the sample solutions was reported with PAD fabricated by 2D patterning techniques [2,35]. Due to the low surface tension and contact angles of solvents with the patterned barrier, it is difficult to hold an excess volume of solvents on filter paper [36,37]. In addition, the liquid samples may contain surfactants that lower the surface energy of solid–liquid–gas interfaces [38]. Liquid samples may preferentially wet the patterned hydrophobic barriers (such as solid wax) and cannot be contained in planar patterns on a filter paper. Moreover, organic solvents and aromatic hydrocarbons may even dissolve the barrier. In either case, they would result in the breaching of the samples [39,40,41]. 

Several approaches have been demonstrated to address the problems of containing liquid samples in PADs. Integrating 3D features in PADs may offer alternative routes to control and regulate the flow of liquid samples in PADs. To this end, paper-based devices with 3D features have been fabricated by stacking layers of patterned papers. Such methods require bonding, aligning, and punching of different substrates [23,42,43,44]. The requirement for the manual processes, however, would increase the complexity of fabrication and may compromise the reproducibility. Materials with high interfacial energy with solvents have been demonstrated to enhance the containment of liquid samples. To this end, barriers based on fluoropolymers were patterned using chemical vapor deposition [45,46]. The fluoropolymer was patterned on filter papers to form circular barriers to contain selected solvents (e.g., ethanol, acetone) for colorimetric assays. Other solvent-resistant materials such as siloxanes [47,48], nail polish [49], pullulan [50], and correction pens [51] were explored as the patterning material in paper-based devices. Alternatively, the containment of solvents may be enhanced by the surface topology of the device. The embossment of a non-woven polypropylene (PP) sheet was demonstrated to create a physical barrier for fluid flow [52]. Polypropylene is resistant to several organic solvents such as isopropyl alcohol (IPA), methanol, and acetone. Overall, the handling and containment of solvent-based samples has been enhanced with (1) alternative materials and (2) device geometry.

To address the issue of solvent containment in PADs, we took a different approach still: FDM 3D printing. FDM 3D printing allows layer-by-layer deposition of thermoplastics on a flat substrate. We hypothesized that the 3D vertical walls integrated with the filter paper would prevent the breaching of the liquids sample through the hydrophobic barriers. Previous work has demonstrated the FDM printing of thermoplastics (polycaprolactone, PCL) on a filter paper followed by the thermal reflow to create planar hydrophobic barriers [53]. To date, however, fabrication of 3D features integrated with PADs using FDM printing has not been achieved. Crucially, the patterned materials need to penetrate through the filter paper by heat reflow while maintaining 3D structures. To achieve that, we patterned two different materials (with different melting points) by multi-material FDM printing: PCL and polylactic acid (PLA). Both PCL and PLA are hydrophobic thermoplastics exhibiting low solubility to selected organic solvents [54,55]. The melting temperatures of thermoplastics depend on their molecular weights and crystalline structures. Previous studies have reported the melting temperatures of 50–60 °C for PCL [56,57,58] and 150–180 °C for PLA [59,60,61,62], respectively. PCL was used to pattern hydrophobic barriers by heat reflow, while PLA was used to print 3D structures. Multimaterial FDM printing permitted seamless connection between PCL and PLA at the interface, and 3D features consisting of PLA were stably integrated with the paper substrate. We fabricated a 3D-PAD with the design of a 96-well plate and studied the compatibility of three common organic solvents (ethanol, IPA, and acetone) in comparison with the 2D PAD fabricated with a solid wax printer. 3D-PAD consisting of PCL and PLA maintained 30 μL of solvents without any leakage. Overall, our method enabled embedding 3D structures on filter paper and should provide opportunities to enhance the analytical capability and functionality of paper-based devices. 

## 2. Experimental Section

### 2.1. Measurement of Viscosity of Polymers

Pellets of the PCL (AliExpress, Hangzhou, China), PLA (Flashforge Corporation, Zhejiang, China), polyethylene (Sigma Aldrich, Singapore, Singapore), and polypropylene (Sigma Aldrich, Singapore, Singapore) were used as purchased. Viscosities of the polymers were measured on a Discovery Hybrid Rheometer HR30 (TA Instruments, New Castle, DE, USA). The TRIOS Express software was set up with a frequency of 1.0 Hz and a temperature ramp rate of 5 °C/min.

### 2.2. Fabrication of 3D-PADs Using a FDM Printer

A total of 9 × 5 wells with an 8.0-mm diameter were designed on AutoCAD (Autodesk, Inc., Mill Valley, CA, USA); the computer-aided design (CAD) of the cylindrical base and wall was converted to a Standard Triangle Language (STL) file for 3D printing. Flashprint slicer and Flashforge Creator Pro (Flashforge Corporation, Zhejiang, China) were used to print the features. The width and height of the wall were 400 μm and 800 μm, respectively. The parameters in Flashprint slicer were: layer height = 0.20 mm, infill density = 100%, printing speed = 40 mm/s, print temperature of PCL = 120 °C, printing temperature of PLA = 180 °C. Before printing on the FDM printer, the laminated filter paper was fixed on the build plate by binder clips to prevent shifting of the paper during the printing. Whatman filter paper was first laminated on the bottom side with the GBC CLA402 laminator (GBC, Lake Zurich, IL, USA). The top side of the laminated filter paper was then placed on the printer bed of Flashforge Creator Pro. The circular base was printed with PCL (AliExpress, Hangzhou, China), and the circular walls were printed with blue PLA (Flashforge Corporation, Jinhua, China). PCL was printed on the filter paper and formed the base of the design, and PLA was printed on PCL. For the penetration of PCL, the printed 3D wells were placed in VWR natural convection oven model VWRU414005-115 (VWR, Singapore, Singapore), and heated at 150 °C for 60 min. The heating temperature in the oven (150 °C) was below the melting temperature of PLA; therefore, the structural integrity of the 3D wells was not compromised.

### 2.3. Fabrication of Planar PADs Using a Solid Wax Printer

Laminating sheets (Aurora, Shanghai, China) were purchased from a local stationery store. Whatman filter paper was first laminated on the bottom side with a GBC CLA402 laminator (GBC, Lake Zurich, IL, USA). A total of 9 × 5 wells with an 8.0 mm diameter were designed using Adobe Illustrator (Adobe Inc., San Jose, CA, USA). A Xerox ColorQube 8580 wax printer (Xerox Corporation, Norwalk, CT, USA) was used to print the circular patterns on the filter paper (Whatman Grade 1, United Scientific Equipment, Singapore, Singapore). The wax-patterned paper wells were heated on a hotplate (Thermo Scientific, Waltham, MA, USA) at 120 °C for 100 s to complete the penetration of the solid wax.

### 2.4. Functionalization of 3D-PAD

The surface of the filter paper was functionalized with 3-aminopropyl triethoxysilane (APTES) (Sigma-Aldrich, Singapore, Singapore). The 3% APTES solution was prepared by diluting APTES with 95% ethanol (Sigma-Aldrich, Singapore, Singapore). An amount of 10 μL of APTES solution was added to each well during salinization, and APTES formed covalent bonds with the hydroxyl groups in cellulose by heating at 110 °C for 30 min. The unbound APTES was washed away with deionized (D.I.) water and dried. The colorimetric reagent, dimethylglyoxime (DMG) (Sigma-Aldrich, Singapore, Singapore) solution was prepared by dissolving in ethanol at 10 mg/mL. DMG molecules were bonded with amino groups on the functionalized surface by heating the 3D-PAD at 95°C for 30 min. The functionalized 3D-PAD was used for the colorimetric assay of Ni (II) ion.

### 2.5. Colorimetric Assay of Nickel (II) Ion

3D-PADs were functionalized with two volumes of DMG solution in ethanol (10 μL and 30 μL, 10 mg/mL) under the process described. Nickel (II) sulfate (NiSO_4_) (Kanto Kagaku, Singapore, Singapore) was prepared at 0.03–20 ppm through the dilution with D.I. water. NiSO_4_ solutions were prepared using 18.2 MΩ·cm ultrapure water (Milli-Q, Millipore, Billerica, MA, USA). An amount of 30 μL of Ni (II) ion solutions were added to the wells, and the formation of pink DMG–Ni (II) complexes was observed. The 3D-PAD was dried in the Binder BD natural convection oven at 60 °C for 15 min. All images for the colorimetric analysis were obtained by placing the 3D-PAD in an A4 carton box. A4 carton box was sprayed with generic black paint and the lid was cut with a small hole for the camera. The images of the 3D-PAD (with varying pink intensities in the wells) were taken using the camera of an iPhone XR (Apple, Cupertino, CA, USA) with default settings and flash enabled. RGB color intensities were separated and analyzed by ImageJ (National Institutes of Health, Bethesda, MD, USA). The Euclidean distances Δ*E* were calculated as follows:

(1) $$\Delta E=\sqrt{{\left(R_{2}-R_{1}\right)}^{2}+{\left(G_{2}-G_{1}\right)}^{2}+{\left(B_{2}-B_{1}\right)}^{2}}$$

where the subscript 1 represented the RGB value of the control zone, and the subscript 2 represented the RGB value of the test zone. The calculated values of Δ*E* were plotted.

## 3. Results and Discussion

### 3.1. Research Scope and Justification

In this research, we proposed to use FDM 3D printing to fabricate 3D structures on a filter paper to enhance the capability to contain solvents. Solid wax printing has been arguably the gold standard in the field of paper-based microfluidics, while the recent discontinuation of solid wax printers (Xerox Corporation, Norwalk, CT, USA) posed difficulty for the fabrication of PADs. It is thus imperative to identify alternative methods to fabricate PADs using readily available instruments. We selected FDM printing as a method to fabricate PADs. FDM printing allowed sequential patterning of multiple polymers that would otherwise require separate processes of patterning, reducing the overall complexity in the fabrication. With the recent expiration of the patent, FDM 3D printers have become relatively inexpensive (<300 USD); a single-nozzle FDM printer would cost less than the solid wax printers. Given the large feature sizes (400-μm width) required in the current study, those low-end FDM printers meet the required specifications. Moreover, FDM filaments are mostly not preparatory and readily available. The availability of the instruments and the materials is crucial to develop the platform technology for the fabrication of low-cost devices.

Fabrication of 3D-PAD requires two types of thermoplastics with the following characteristics. Firstly, both thermoplastics must be made into the filamentous form to pattern by FDM printing. Secondly, two thermoplastics should possess sufficiently different melting points—one for the molten reflow and one for the fabrication of 3D features. Thirdly, the base material (i.e., the material used to create hydrophobic barriers by the molten reflow) should have relatively low viscosity (<1000 Pa·s) to ensure quick penetration through the paper. Lastly, both materials should be compatible with some common solvents. To this end, we have characterized the viscosities of four common thermoplastics applicable for FDM printing (Figure S1).

While most of the FDM-compatible polymers have melting temperatures of >150 °C, PCL has a relatively low melting point (50–60 °C). The low viscosity of PCL (~390 Pa·s, 150 °C) suggested the feasibility to perform molten reflow through the filter paper (Whatman Grade 1, 11-μm average pore sizes and 180-μm thick). PLA is a widely available material that is commonly used in the FDM printer and has a higher melting temperature of ~170 °C. It is compatible with some alcohols and aromatic hydrocarbons [54] and was used to create a physical wall on the PCL base material. We hypothesized that the presence of 3D features should enhance the solvent containment as compared to the 2D barriers that solely depend on the interfacial energies between the fluid samples and the patterned materials.

### 3.2. Fabrication of 3D-PAD

**Figure 1:**
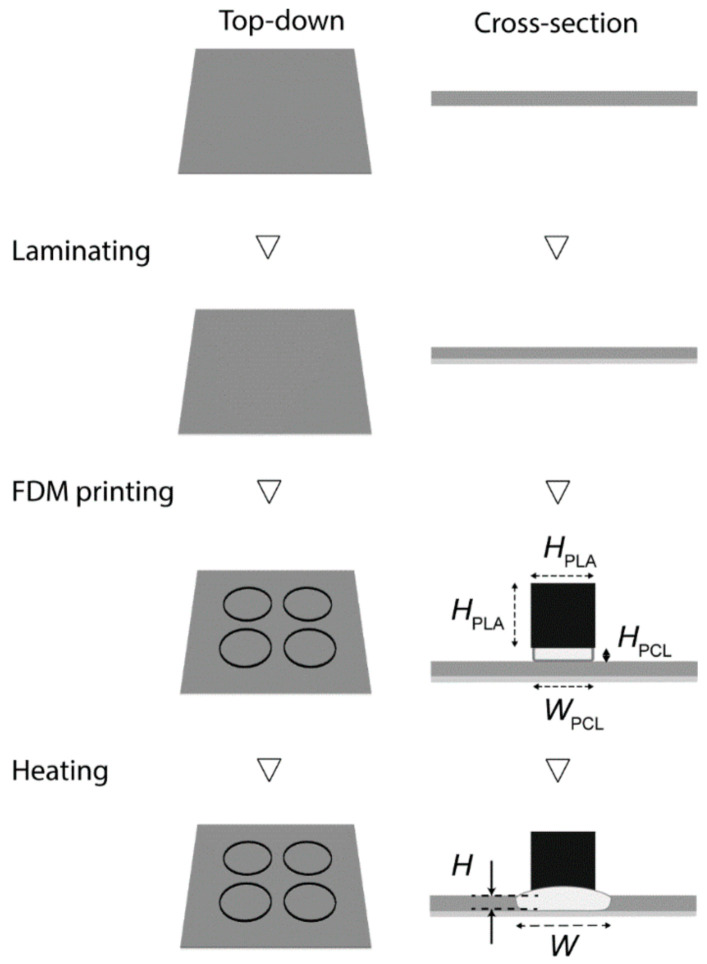
Schematic illustration of the fabrication of 3D-paper-based analytical devices (PAD). Firstly, the filter paper was laminated on the bottom side. The circular base was then printed with polycaprolactone (PCL), and the wall was printed with polylactic acid (PLA). The entire device was placed in the oven and heated at 150 °C for 60 min for the thermal reflow of PCL.

Figure 1 illustrated the fabrication of 3D-PAD. We first designed the 3D structures consisting of two parts: the base and the wall. The base was PCL that penetrated through the paper, and the wall was PLA that maintained the printed structure. The filter paper was laminated with the lamination film (made of polyethylene terephthalate, PET) at the bottom side to prevent the flow of liquids. The laminated paper was then patterned with two thermoplastics using an FDM printer. PCL was printed first and PLA was printed on the top of PCL. After printing, the patterned filter paper was placed in an oven to permit the reflow of PCL through the filter paper. The height (*H*) and the width (*W*) of the PCL changed as they were heated. We characterized *H* and *W* to identify the condition to fabricate 3D-PAD.

To determine the temperature and duration of the reflow of PCL, we measured the viscosity of molten PCL. The viscosity of the molten PCL is temperature-dependent, where the viscosity decreases at higher temperatures. The viscosity of molten PCL was measured as 1130 Pa·s at 100 °C and 390 Pa·s at 150 °C. The penetration of PCL was approximated as the capillary flow through the micropores of the filter paper. According to the Lucas–Washburn equation for the capillary flow in the porous material, the rate of penetration of PCL is inversely proportional to the square root of the viscosity [63]. Under this assumption, the heating time would be 1.7 times longer at 100 °C than at 150 °C. Although the increase in the temperature would further decrease the heating time, we selected the heating temperature of 150 °C to keep PLA unmolten (melting point (m.p.) ~ 150–180 °C). We experimentally confirmed that heating at 150 °C did not change the printed PLA structure. 

**Figure 2:**
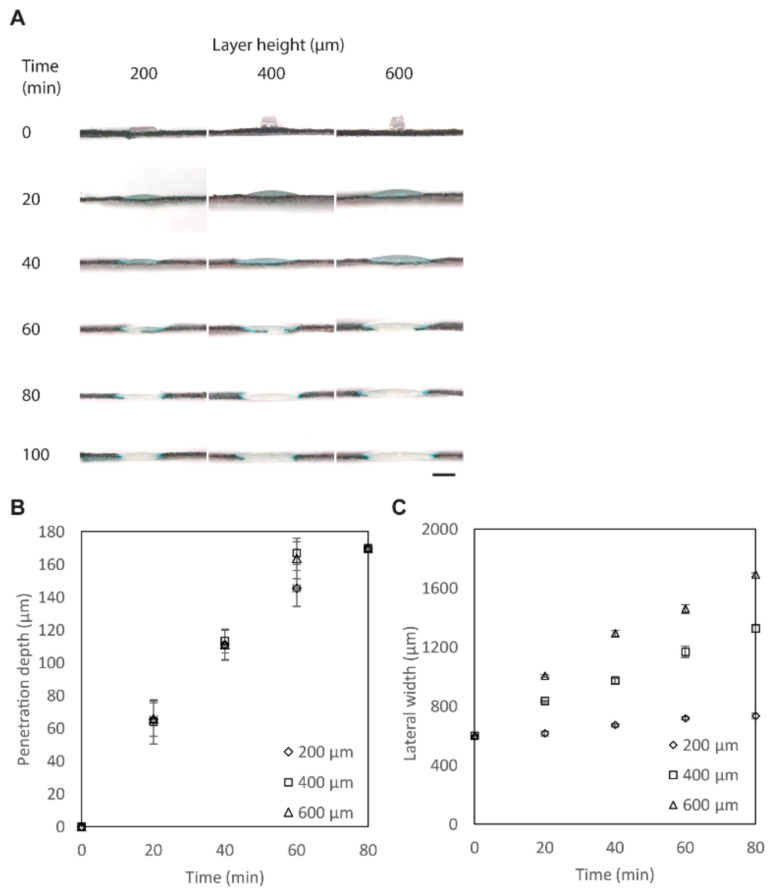
Penetration of PCL through the filter paper. (**A**) Optical micrographs of the cross-sections of the filter paper printed with PCL. The paper was dyed with black ink to highlight the location of the penetrated polymers. Scale bar = 800 μm. (**B**) Plot showing the penetrated height of PCL (*H*) (*H*_PCL_ = 200, 400, and 600 μm) heated at 150 °C at different timpoints (n = 4). (**C**) Plot showing the lateral widths of PCL (*W*) (*H*_PCL_ = 200, 400, and 600 μm) heated at 150 °C at different timepoints (n = 4).

To understand the time required for the penetration of PCL, PCL was patterned on filter paper (laminated with a PET film) with *H*_PCL_ = 200, 400, and 600 μm and *W*_PCL_ = 400 μm. The lateral spreading and the penetration of PCL on filter paper were characterized after heating in the convection oven at 150 °C for a duration of up to 100 min (Figure 2A). With a height of 200 μm, the patterned PCL took 80 min to penetrate through the entire thickness of the filter paper (~180 μm). With heights of 400 and 600 μm, the patterned PCL took 60 min to penetrate through the same filter paper (Figure 2B). PCL also spread laterally. The initial widths of the printed PCL were ~600 μm while the pattern was designed as *W*_PCL_ = 400 μm; the increase in the width in the first layer was inevitable because the molten PCL was directly patterned on the cellulose paper. As the height of the PCL was increased, the lateral width (*W*_L_) after heating also increased (Figure 2C). Overall, we selected *H*_PCL_ = 400 μm because the full vertical penetration was achieved at 60 min while the lateral spreading was not as prominent as *H*_PCL_ = 600 μm.

**Figure 3:**
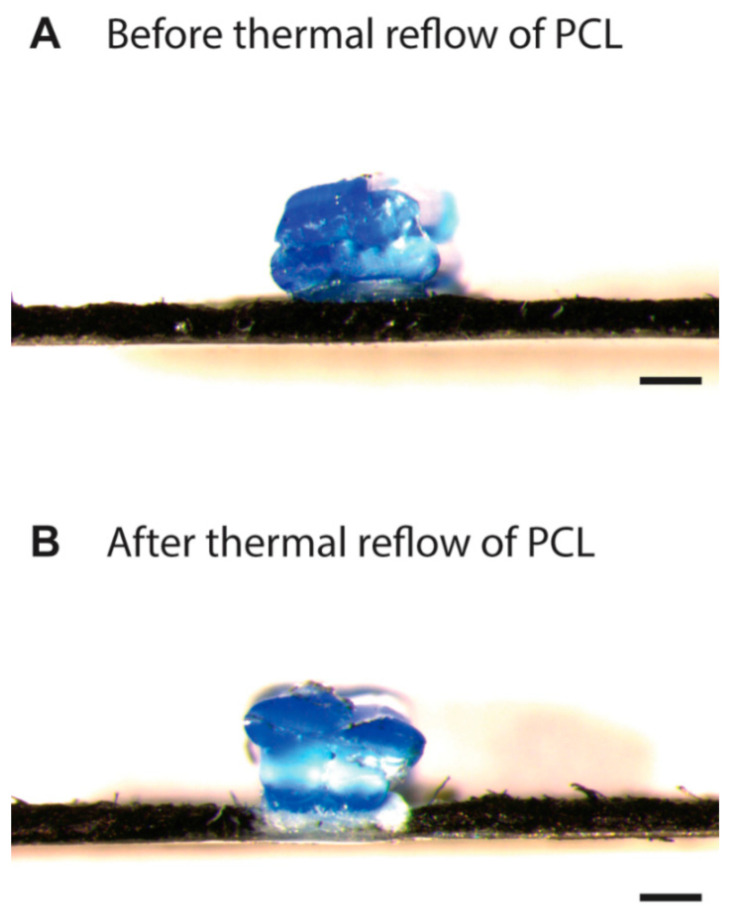
(**A**) Cross-sectional image of PCL and PLA printed on filter paper using an fused deposition modeling (FDM) printer before the thermal reflow. The layer of PCL was partially melted by the deposition of molten PLA from the top. (**B**) Cross-sectional image of PCL and PLA printed on filter paper after heating in an oven at 150 °C for 60 min. The filter paper was dyed with black ink to enhance the contrast, and the penetration of the PCL was highlighted. Scale bars = 400 μm.

Once we identified the conditions for the penetration of PCL, we fabricated 3D wells on a filter paper using PLA. PCL (*H*_PCL_ = 400 μm, *W*_PCL_ = 400 μm) and PLA (*H*_PLA_ = 800 μm, *W*_PLA_ = 400 μm) were printed on a filter paper laminated with a PET film underneath. The cross-sectional image shows that PCL did not penetrate through the filter paper before heating (Figure 3A; the filter paper was dyed in black to enhance the contrast). We observed that the extrusion of molten PLA at 180 °C partially melted the surface of the PCL underneath. The entire sample was then heated in an oven at 150 °C for 60 min for thermal reflow of PCL. After heating, the PLA and PCL were seamlessly connected, and the printed PLA could not be mechanically peeled off. The penetration of PCL was completed as apparent from the white space in the filter paper blocking the breeching of the black dye (Figure 3B).

### 3.3. Print Speed and Limitation 

The print speed of FDM 3D printing is generally lower than that of 2D printing (e.g., solid wax printing and inkjet printing). Depositing the materials for a 96-well plate (i.e., 8.5 cm × 11.5 cm) with the set dimensions of the filaments (*H*_PCL_ = 400 μm, *W*_PCL_ = 400 μm, *H*_PLA_ = 800 μm, and *W*_PLA_ = 400 μm) requires ~8 min by FDM 3D printing. Patterning the materials for the same device with solid wax printing requires <1 min. Because of the difference in the printing mechanisms, the fabrication of the device with the same footprint inevitably takes longer in FDM 3D printing than 2D printing. Nevertheless, the developed methods shall be useful when the application requires 3D features integrated with PADs, which are difficult to attain by other methods of fabrication. The achievable size of the device depends on the build volume of the 3D printer to be used. In the current demonstration, the build volume was 23 cm × 15 cm × 15 cm (width × length × height). The lateral width of the PCL increased from 400 μm to 1200 μm by heating, which must be taken into consideration when designing the device.

### 3.4. Ethanol, IPA, and Acetone in 3D-PAD

**Figure 4:**
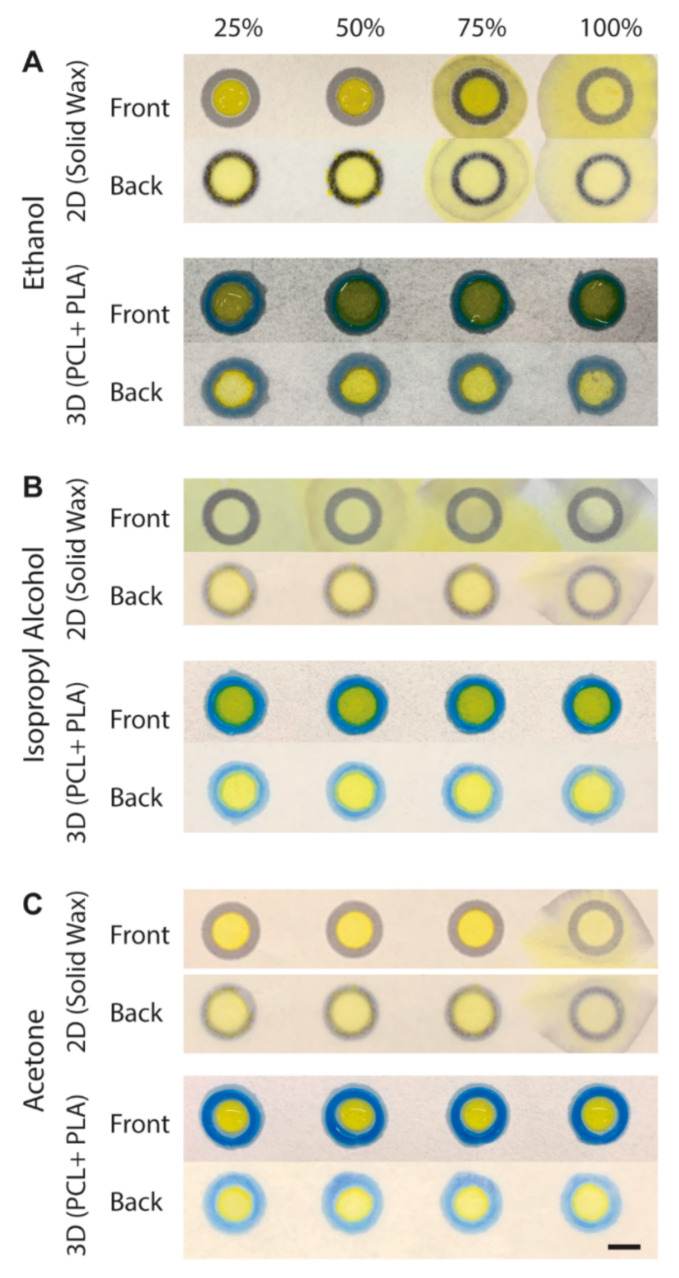
Solvent containment in 3D-PAD. An amount of 30 μL of 25–100% of (**A**) ethanol (**B**) isopropyl alcohol (IPA) and (**C**) acetone placed on 2D wax wells and 3D wells. Both front and back sides of the device are shown. Scale bar = 400 μm.

Hydrophobic or non-polar molecules are often insoluble in water and require organic solvents to dissolve them. Ethanol, IPA, and acetone are common solvents for dissolving such compounds. To demonstrate the capability of the 3D-PAD to hold solvents, we compared the performance of solvent containment (30 μL) in the 2D wells printed by wax printing of the same diameter (*d* = 8.0 mm) and in the 3D-PAD. Three solvents (ethanol, IPA, and acetone) were diluted with D.I. water to 25%, 50%, 75% (by volume); together with the undiluted solvents (100%), three solvents at four different concentrations were placed in the 2D wax wells as well as the 3D wells. Yellow inkjet dye was added to the solvents to enhance the contrast. For ethanol, two solvents (75% and 100%) breached the 2D wax wells, while all solvents were maintained in the 3D wells (Figure 4A). For IPA, all solvents (25%, 50%, 75%, and 100%) breached the 2D wax wells, while all solvents were maintained in the 3D wells (Figure 4B). For acetone, pure acetone (100%) breached the 2D wax wells, while all solvents were maintained in the 3D wells (Figure 4C). These experiments suggested that (1) the 2D wax wells do not readily isolate the liquid samples containing organic solvents, and (2) the 3D wall consisting of PLA enhanced the containment of the solvents. We note that PLA was partially soluble in acetone, while the short duration (<3 min) to enclose 30 μL of acetone in the 3D wells did not affect the structural integrity of the 3D-PAD.

### 3.5. 3D-PAD for Ni (II) Ion Analysis

Dimethylglyoxime (DMG) is a colorless chemical that produces a deep pink color when it forms a complex with Ni (II) ion. To perform the colorimetric assays in PAD, DMG can be immobilized to the filter paper using 3-aminopropyl triethoxysilane (APTES). DMG is insoluble in water and must be dissolved in ethanol to carry out the bonding of DMG to APTES for colorimetric detection of Ni (II). The analysis of Ni (II) ion was previously performed using a 2D well on a filter paper fabricated by chemical vapor deposition of a fluoropolymer [46]. Although the use of fluoropolymer enhanced the solvent containment, 2D wells did not permit containment of a large volume of solvents; the volume of the DMG solution in ethanol was 2 μL, and the volume of the analytes (Ni (II) ion) was 10 μL [46]. In 3D-PAD we developed, the vertical wells provided physical barriers to contain liquid materials. As demonstrated, 30 μL of ethanol-based solutions can be readily contained in the well, which was crucial in enhancing the sensitivity of Ni (II) assay using DMG on a filter paper.

**Figure 5:**
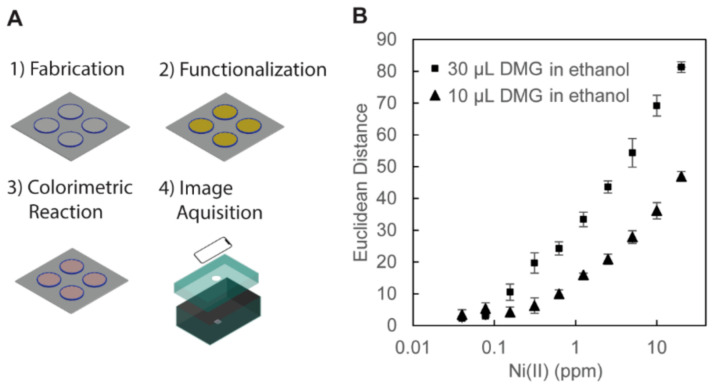
(**A**) Illustration of the procedures for the colorimetric assay of Ni (II) ion. (1) Fabrication: the 3D-PAD was fabricated by FDM printing. (2) Functionalization: the surface of the wells of the 3D-PAD was functionalized with 3-aminopropyl triethoxysilane (APTES) and dimethylglyoxime (DMG) dissolved in ethanol. (3) Colorimetric Reaction: aqueous solutions of Ni (II) ion were added in the 3D wells and dried in an oven, and pink DMG–Ni (II) complexes were formed. (4) Image Acquisition: the device was placed in a blackened carton box for the observation. The images of the 3D-PAD were taken with an iPhone XR camera for analysis. (**B**) Plots showing the color intensity of DMG-Ni (II) complexes with respect to the concentration of Ni (II) ions (with the log scale in the horizontal axis). Two volumes of DMG in ethanol (30 μL and 10 μL, 10 mg/mL) were tested to functionalize the cellulose in the 3D wells. The colors of the well on the 3D-PAD were measured from the RGB values of the acquired images (n = 4).

To demonstrate the colorimetric detection of Ni (II) ion (Figure 5), we first immobilized APTES to the hydroxyl groups in the cellulose. DMG was then covalently bonded to the amino group of immobilized APTES by heating the device at 95 °C for 30 min. Two volumes of DMG in ethanol (10 μL and 30 μL, 10 mg/mL) were tested to demonstrate different degrees of surface functionalization by the DMG solutions. Then, the solution containing Ni (II) ion was added to the well to perform colorimetric assays. A pink DMG–Ni (II) complex was formed in the presence of Ni (II) ion. We measured the Euclidean distance of the color data extracted from the RGB values. The wells treated with 30 μL of DMG in ethanol exhibited higher color intensities at all concentrations of Ni (II) ions than the wells treated with 10 μL of DMG in ethanol (Figure 5B). In the previous work, the functionalization of the filter paper with DMG was performed with 2 μL of DMG in ethanol (10 mg/mL, the same concentration as the current experiment). We estimated the limit of detection (LOD) as 3.3 σ/S, where σ is the standard deviation and S is the gradient of the tangential line [64]. The calculated LOD of Ni (II) ions in the 3D wells (functionalized with 30 μL of DMG in ethanol) was 0.129 ppm, which was lower than 0.24 ppm in the previous demonstration [46]. This demonstration suggested that the presence of the vertical walls permits the use of an increased amount of solutions (e.g., ethanol), which contributed to enhancing the sensitivity of colorimetric assays on PADs.

## 4. Conclusions

We demonstrated the fabrication of 3D wells in paper analytical devices (PAD), which we termed 3D-PAD. Multi-material FDM printing enabled fabrication of 3D structures on a filter paper that were seamlessly connected to the hydrophobic barriers embedded in the paper. The combination of the two polymers (e.g., PCL and PLA) with a large difference in the melting points allowed fabrication of such structures. We characterized the depth of penetration and the lateral spreading of PCL on a filter paper and identified the parameters to fabricate the device. 3D-PAD was able to hold a large volume (30 μL) of ethanol, IPA, and acetone without leakage. As a demonstration, we performed a colorimetric assay of Ni (II) ion in 3D-PAD, where DMG in ethanol was used to functionalize the paper substrate. The use of 3D wells permitted handling of an increased amount of liquid reagents and samples, which then improved the sensitivity of the colorimetric assays.

With the discontinuation of the commercial solid wax printers, FDM printing is a promising candidate as the tool to perform rapid prototyping of PAD. We demonstrated that FDM offered an alternative route to pattern hydrophobic materials on cellulose papers. Our demonstration also highlighted the importance of using multiple materials for the fabrication of 3D features on PADs: a base material (e.g., PCL) to create hydrophobic barriers on a filter paper, and an additional material (e.g., PLA) to create 3D features integrated with the base material. While the current work demonstrated the use of 3D features to enhance the containment of the solvent, we believe such 3D features integrated with PAD enable advanced handling of liquid samples (such as a 3D fluidic reservoir, a flow regulator, and a reaction chamber) to unlock the full potential of paper-based analytical devices.

## Data Availability

Data is contained within the article or supplementary materials.

## Supplementary Materials

The following are available online at https://www.mdpi.com/2079-6374/11/3/84/s1, Figure S1: Plot showing the viscosities of four FDM-printable polymers—poly(caprolactone) (PCL), poly(lactic acid) (PLA), polyethylene (PE), poly(propylene) (PP)—with respect to the temperature.
