# Supplementary material for: 3D-PAD: Paper-Based Analytical Devices with Integrated Three-Dimensional Features
Source: Biosensors (Basel). 2021 Mar 17;11(3):84. doi: 10.3390/bios11030084 (PMC8002416; doi:10.3390/bios11030084)
Supplement: Supplementary file 1 [file biosensors-11-00084-s001.pdf]

Communication

3D-PAD: Paper-Based Analytical Devices with Integrated Three-Dimensional Features

James S. Ng [1] and Michinao Hashimoto [1,2,3,*]

[1] Pillar of Engineering Product Development, Singapore University of Technology and Design, 8 Somapah Road, Singapore 487372, Singapore; james_ng@mymail.sutd.edu.sg
[2] SUTD-MIT International Design Centre, Singapore University of Technology and Design, 8 Somapah Road, Singapore 487372, Singapore
[3] Digital Manufacturing and Design Centre, Singapore University of Technology and Design, 8 Somapah Road, Singapore 487372, Singapore
* Correspondence: hashimoto@sutd.edu.sg; Tel.: +65-6499-4867

Supplementary

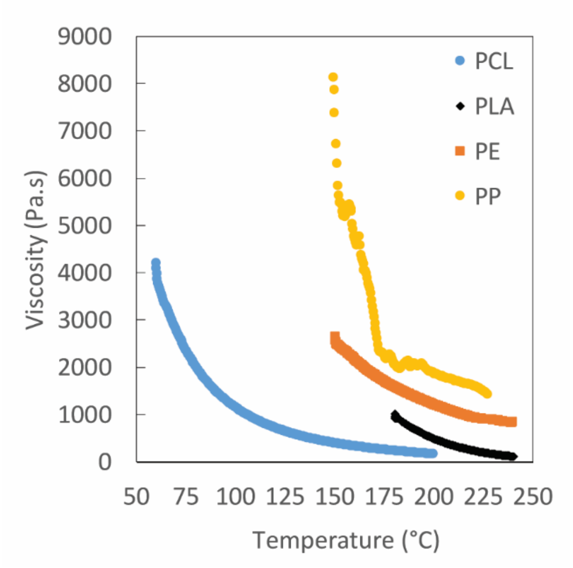

Figure S1. Plot showing the viscosities of four FDM-printable polymers—poly(caprolactone) (PCL), poly(lactic acid) (PLA), polyethylene (PE), poly(propylene) (PP)—with respect to the temperature.
